# Evaluation of the pSMILE (Palliative care-Situational Motivating Interactive Learning and Education) educational program based on web-based and face-to-face workshops for palliative care pharmacists

**DOI:** 10.1186/s40780-025-00491-w

**Published:** 2025-12-20

**Authors:** Megumi Kabeya, Mayako Uchida, Masahiko Amagawa, Makio Imamura, Yoko Kasahara, Yasunori Miyamoto, Takuya Yano, Takayuki Nakagawa

**Affiliations:** 1 Department of Pharmacy, Nagoya Memorial Hospital, 4-305 Hirabari, Tempaku-Ku, Nagoya, Aichi 468-8520 Japan; 2 Department of Clinical Pharmacology and Therapeutics, Kyushu University Hospital, 3-1-1, Maidashi, Higashi-Ku, Fukuoka, Fukuoka, 812-8582 Japan; 3 Department of Pharmacy and Cooperation, Palliative Care Clinic Ippo, 790 Kyome-Machi, Takasaki, Gunma 370-0011 Japan; 4 Department of Clinical Support, Kurashiki Medical Center, 250 Bakuro-Cho, Kurashiki, Okayama 710-8522 Japan; 5 Department of Pharmacy, Akimoto Clinic, Hiroshima, 3-34 Inari-Machi, Kaita-Cho, Aki-Gun, Hiroshima, 736-0067 Japan; 6 Department of Pharmacy, Hamamatsu Medical Center, 328 Tomitsuka-Cho, Chuo-Ku, Hamamatsu, Shizuoka, 432-8580 Japan; 7 Department of Pharmacy, Sumitomo Besshi Hospital, 3-1 Oji-Cho, Niihama, Ehime, 792-8543 Japan; 8 Department of Clinical Pharmacology and Pharmacotherapy, Wakayama Medical University, 25 Shichibancho, Wakayama, 640-8156 Japan; 9 Education and Training Committee, Japanese Society for Pharmaceutical Palliative Care and Sciences, Tokyo, Japan

**Keywords:** Palliative care, Behavioral changes, Pharmacist education, pSMILE, Japanese Society for Pharmaceutical Palliative Care and Sciences, Web-based workshop

## Abstract

**Background:**

In 2021, the Education and Training Committee of the Japanese Society for Pharmaceutical Palliative Care and Sciences established an educational training program for pharmacists in palliative care, named Palliative care-Situational Motivating Interactive Learning and Education (pSMILE), based on web-based and face-to-face workshops. The program aims to enhance pharmacists’ knowledge of palliative care and develop communication and cooperation skills.

**Methods:**

We constructed a web-based pSMILE training program, which was later adapted for face-to-face workshops. This program consisted of two sessions, with the first half at a hospital and the second half at a clinic or community pharmacy. The participants could choose from three learning scenarios. A post-survey (within 1 week of the workshop) assessed usefulness, difficulty, length, and satisfaction. Participants also completed a web-based survey on behavioral changes related to daily palliative care following the workshop. Nine items on behavioral changes were assessed before and 1 month after the workshop, which was held from April 2021 to March 2024.

**Results:**

Twelve pSMILE workshops were held during this period (10 web-based and two face-to-face). A total of 296 pharmacists participated, 152 (51.4%) of whom were Board Certified Pharmacist in Palliative Pharmacy, and 292 (98.6%) responded to the before/after workshop survey. Usefulness ratings were 4.39 for Session I and 4.20 for Session II. Satisfaction ratings were high (≥ 4.5), with no significant differences based on affiliation, training format, or certification. All confidence scores of nine daily palliative care behaviors (symptom assessment; multidisciplinary information sharing; proposing pharmacotherapy; polypharmacy intervention; palliative pharmacotherapy with awareness of pharmacokinetics; explanation of delirium; response in the discharge conference; information sharing between hospitals, clinics, and community pharmacies; and pharmacotherapy suggestions in view of the post-discharge) improved significantly post-workshop (*p* < 0.01), across both web-based and face-to-face workshops. Certified participants had higher confidence scores both before and after the workshop, and each group showed a significant improvement.

**Conclusions:**

These results suggest that either web-based or face-to-face pSMILE workshops improve the quality of pharmacists’ contribution in daily palliative care. This is the first report on the effectiveness of an academically approved web-based educational program for palliative care pharmacists, comparable to face-to-face workshops.

**Supplementary Information:**

The online version contains supplementary material available at 10.1186/s40780-025-00491-w.

## Background

Palliative care should be provided from the time of cancer diagnosis and simultaneously with cancer treatment [1, 2]. Compared to cancer treatment alone, early palliative care is reported to improve quality of life, symptoms, and patient-clinician communication [3–5]. Palliative care should support patients with cancer and their families across all stages of the disease, not only at the end of life but also by addressing both physical and psychosomatic distress. Guidelines regarding the role of pharmacists in palliative care have been established [6], and their central role is to support symptom management and pharmacotherapy [7, 8].

Symptom relief through collaboration among multiple professionals is necessary to decrease distress in patients [1]. Multi-professional cooperation is important for palliative care, but effective team medicine cannot be achieved simply by bringing together multiple professionals. There must be a common understanding of their roles and responsibilities, as well as an attitude of mutual respect. Effective communication between healthcare providers and patients is also essential [9, 10]. A strong positive relationship has been shown between the communication skills of healthcare providers and the patient’s receptivity to the proposed medical action [11], which increases patient satisfaction and improves outcomes [10, 12]. Therefore, pharmacists are required to acquire high communication skills in addition to specialized knowledge and skills for pharmacotherapy [7].

Previous education and training workshops hosted by the Japanese Society for Pharmaceutical Palliative Care and Sciences (JPPS), called as the Pharmacy Education for Oncology and Palliative care Leading to happy End-of-life (PEOPLE) program, have been held face-to-face several times per year in various cities to enhance communication skills of palliative care pharmacists. However, in 2020, almost all workshops and conferences were cancelled because of the Covid-19 pandemic in Japan. To resolve these problems, we discussed the possibility of organizing a communication training program that can be held without close contact. In addition, we have been seeking a training program that is easily accessible to pharmacists, not only in cities, but also in rural areas. Therefore, we organized a training program in web-based workshops with the approval of the JPPS. The Education and Training Committee established a new web-based training course in 2021 named the Palliative care-Situational Motivating Interactive Learning and Education (pSMILE) workshop for pharmacists in palliative care. ‘pSMILE’ embodies the intention and hope “to provide quality palliative care in any situation while actively interacting with people in various positions” and “to bring a smile to as many patients and their families as possible through the activities of pharmacists.” pSMILE, as the successor to the PEOPLE program, has been enhanced in learning diversity, session focus, time efficiency, and academic depth. From April 2023, face-to-face on-site workshops were resumed as the infection situation was calming, and both web-based and face-to-face workshops were held as pSMILE programs.

In addition to providing knowledge about palliative care, the pSMILE training program aims to equip participants with communication skills and attitudes in collaboration with other professions and facilities. In workshops, it is important not only to convince and satisfy participants but also to put them into practice in their daily work in palliative care. Thus, in this study, we evaluated the effectiveness and usefulness of the pSMILE training program by focusing on the behavioral changes in participants in daily palliative care through pre- and post-surveys. This is the first report to show the effectiveness and usefulness of web-based workshops, as well as on-site face-to-face workshops, in improving the communication skills and attitudes of palliative care pharmacists.

## Methods

### Program of pSMILE

This program is designed for pharmacists engaged in palliative care in hospitals, community pharmacies, or clinics to provide in-depth knowledge about palliative care, as well as communication skills and attitudes in cooperation with other professions and facilities. We first constructed the main training to be held web-based using a web conferencing system (Zoom) but later adapted it to face-to-face workshops on site. The program consisted of two sessions, with the first half being in a hospital setting and the second half in a clinic or community pharmacy setting. The web-based and face-to-face workshops used the same pre-assignments and case scenarios, ensuring consistency in the program content. The main differences lay in their duration. The duration of web-based workshops was shortened to 4 h, making it easier for participants to attend as they could be completed within a half day. In contrast, the face-to-face workshops lasted 7 h, and allocated approximately 1.5 times more than the web-based workshops for ice breaking, small group discussion, presentation, explanation and comment to promote interaction among participants (Table 1). Members of the Education and Training Committee of the JPPS were responsible for drafting the basic pSMILE program, discussing details such as specific times and methods, and completing the program. In developing the educational program, we referred to the American Society of Health-System Pharmacists (ASHP) Guidelines on the Pharmacist’s Role in Palliative and Hospice Care [6]. The guideline categorizes pharmacists’ activities into essential and desirable roles (Supplementary Table 1). Based on this framework, the pSMILE workshop was designed to ensure that participants acquire the essential competencies required for daily palliative care practice (e.g., symptom assessment, information sharing, pharmacotherapy proposals), while also providing opportunities to engage with desirable roles (e.g., participation in multidisciplinary teams, leadership, and education). The committee asked the Board of Directors of JPPS to review the pSMILE program, which was then approved.

**Table 1:** Program of web-based and face-to-face pSMILE workshops

Program		Web-based (4 h)	Face- to-face (7 h)
Opening		5 min	10 min
Ice breaking		10 min	20 min
Session Ⅰ	Small group discussion	60 min	90 min
	Presentation	25 min	30 min
	Explanation and comment	20 min	30 min
Break time		10 min	60 min (lunch time)
Session Ⅱ	Small group discussion	60 min	90 min
	Presentation	25 min	30 min
	Explanation and comment	20 min	30 min
Closing		5 min	30 min

Three learning scenarios have been developed. All authors repeatedly discussed the training learning scenarios, checked for any problems, including updates of explanations, and revised them according to requests from the survey before each pSMILE workshop. Table 2 lists the learning content and objectives from April 2021 to March 2024. All lecturers and facilitators attended the pSMILE facilitator training course and received training.

**Table 2:** Learning objectives and contents in three situations

Situation	Scenario 1 (since July 2021)	Scenario 2 (since February 2023)	Scenario 3 (since February 2024)
Session Ⅰ	Inpatient palliative case (Hospital pharmacists)	Outpatient palliative case during chemotherapy (Hospital pharmacists for outpatients)	Outpatient treatment task share case (Hospital pharmacist for outpatients – Community pharmacy)
Session Ⅱ	Community collaboration case in discharge conference (Hospital and community pharmacy pharmacists)	Home palliative case (in-home medical care management guidance, how to meet the end of life)	Home palliative case (Transition to home, prescribing practice)
Learning Objectives	As a case study in palliative care for inpatients with cancer, the participants discuss about pain relief, devising suggestions to other professions and points for improvement in hospital, and develop the ability to put them into practice. In addition, assuming a discharge conference, participants will learn how to cooperate among facilities and with other professions	As a case study in palliative care for an outpatient with cancer during chemotherapy, the participants discuss an assessment, proposal of symptomatic methods with the attending physician, the details of contact with the community pharmacy as a pharmacist in charge of outpatient, and develop the ability to put them into practice. In addition, assuming the transition from the pharmacy counter at the community pharmacy to in-home medical care management guidance, the participants will discuss symptomatic methods to alleviate pain and learn how to meet the end of life, as well as how to cooperate with other professions	As a case study in palliative care for outpatients with cancer, the participants develop the ability to practice outpatient treatment task sharing by proposing prescriptions to physicians after assessing the side effects of anticancer drugs and pain relief as a pharmacist in charge of outpatient or community pharmacy In addition, the participants learn how to collaborate with other professions by considering how patients and their families should spend their final days, as well as proposing prescriptions for injectable drugs, while considering their thoughts and feelings

### Pre- and post-survey for pSMILE workshop

Pharmacists who participated in the pSMILE training program were asked to complete a web-based pre- and post-survey for the pSMILE workshop (before and within 1 week after pSMILE participation, respectively). To avoid duplication, only the first participation of each pharmacist was included in the present analyses. The respondents were not offered compensation for their participation. The pre-survey assessed participants’ characteristics such as age, years of experience as a palliative care pharmacist, place of work, place of residence, and certification in palliative pharmacotherapy. On the day of the workshop, groups of participants were equally divided based on the results of the pre-survey. The post-survey assessed the usefulness, difficulty, length, and satisfaction of the pSMILE workshop (Table 3). Questions 1 and 4 assessed the usefulness of the sessions in daily work. Questions 2 and 5 pertained to the difficulty level of the sessions. Questions 3 and 6 asked about the duration of each session. Question 7 asked about participants’ satisfaction with the workshop after completing the pre-assignment. Question 8 asked about satisfaction with the web-based training format. Question 9 asked about the overall satisfaction with the workshop. They were asked to respond to each questionnaire on a 5-point scale from 1 (not at all) to 5 (very much) for questions 1 and 4; from 1 (very easy) to 5 (very difficult) for questions 2 and 5; from 1 (very short) to 5 (very long) for questions 3 and 6; and from 1 (dissatisfied) to 5 (satisfied) for questions 7–9.

**Table 3:** Questionnaire survey within 1 week after pSMILE participation

No	Contents of questionnaire	Scores
Q1	Are you able to apply the content of Session I in your daily work?	from 1 (not at all) to 5 (very much)
Q2	How difficult did you find the content of Session I?	from 1 (very easy) to 5 (very difficult)
Q3	How appropriate was the length of Session I for you?	from 1 (very short) to 5 (very long)
Q4	Are you able to apply the content of Session II in your daily work?	from 1 (not at all) to 5 (very much)
Q5	How difficult did you find the content of Session II?	from 1 (very easy) to 5 (very difficult)
Q6	How appropriate was the length of Session II for you?	from 1 (very short) to 5 (very long)
Q7	What are your thoughts on completing pre-assignments before attending pSMILE?	from 1 (dissatisfied) to 5 (satisfied)
Q8	How satisfied are you with the web-based training format?	from 1 (dissatisfied) to 5 (satisfied)
Q9	How satisfied are you overall with pSMILE in this context?	from 1 (dissatisfied) to 5 (satisfied)

### Survey for behavioral changes related to daily palliative care following pSMILE program

Pharmacists who participated in the pSMILE training program were asked to complete a web-based survey on behavioral changes following the workshop. Behaviors related to daily palliative care were assessed at two time points: before and 1 month after the workshop. Analyses were conducted anonymously. No compensation was offered to the respondents for the survey. However, completion of the survey was part of the training program and a requirement for credit granting. Table 4 shows the tasks related to behavioral changes. The tasks included nine items (symptom assessment; multidisciplinary information sharing; proposing pharmacotherapy to the attending physician; polypharmacy intervention for patients undergoing palliative treatment; palliative pharmacotherapy practice with awareness of pharmacokinetics; explanation of delirium symptoms to patients and their families; responses at the time of participation in the pre-discharge conference; information sharing between hospitals, clinics, and community pharmacies; and suggestions for pharmacotherapy in view of the post-discharge period). Participants were asked to rate their level of confidence in each task on an 11-point scale ranging from 0 (no confidence at all) to 10 (very confident).

**Table 4:** Questionnaires on behavioral changes in palliative care before and 1 month after pSMILE training

	Palliative care tasks
a	Symptom assessment considered from multiple perspectives
b	Information sharing with other multiple professions
c	Suggestion of drug therapy to the attending physician
d	Polypharmacy intervention for patients undergoing palliative treatment
e	Practice of palliative drug therapy with consideration of pharmacokinetics
f	Explaining delirium symptoms to patients and families
g	Responses when participating in a discharge conference
h	Information sharing between hospitals, clinics, and community pharmacies
i	Proposals for drug therapy based on life after hospital discharge

These tasks were scored from 0 (no confidence) to 10 (very confident) on an 11-point scale

### Statistical analysis

Data were analyzed using descriptive statistics. Distributions of sex, training format, case scenario, and certification status were analyzed using Fisher’s exact test. Age was compared using one-way analysis of variance (ANOVA). Comparison of scores from the three groups (clinic, hospital, and community pharmacy pharmacists) was performed using one-way ANOVA, followed by Tukey’s honest significant difference test (Tukey’s HSD test). Statistical analyses between the two groups (web-based vs. face-to-face workshops, and with vs. without certification in palliative pharmacotherapy) were performed using *t*-tests. Scores were compared using a paired *t*-test to confirm behavioral changes before and after the pSMILE workshop. All statistical analyses were performed using EZR (Saitama Medical Center, Jichi Medical University, Saitama, Japan) [13] and R version 4–4-1 (R Core Team, 2023) with the packages of “ggpubr” for the graphics. All *p*-values were two sided, and *p*-values of 0.05 or less were considered statistically significant. Data are presented as the mean ± standard deviation.

## Results

From April 2021 to March 2024, 334 pharmacists participated in the pSMILE program. Of these, 4 pharmacists attended three times, and 27 attended twice. To avoid duplication, only the first participation of each pharmacist was included in the analyses, resulting in 299 participants. Among them, 296 participants (99%) responded to the pre- and post-survey within 1 week after the workshop, whereas 292 (97.7%) responded to the behavioral change survey 1 month after the workshop. We analyzed the answers from 296 respondents regarding the characteristics of the respondents and assessments of the workshop, as well as from 292 respondents regarding the behavioral changes of participants in the palliative care tasks following the workshop.

### Characteristics of respondents

The characteristics of respondents are listed in Table 5. The respondents comprised 217 (73.3%) hospital, 74 (25%) community pharmacy, and 5 (1.7%) clinic pharmacists. There were 167 (56.4%) males and 129 (43.6%) females, with no significant difference in facility affiliation (clinic, hospital, or pharmacy). Similarly, no significant difference was found in the distribution of training formats (web-based vs. face-to-face) by facility affiliation. The number of certified and non-certified respondents was almost the same: 152 (51.4%) Board Certified Pharmacist in Palliative Pharmacy (BCPPP) and 144 (48.6%) non-BCPPP. By contrast, certification rates differed significantly by facility affiliation (*p* < 0.001), with the highest in clinic (80%), followed by hospital (60.8%), and the lowest in the pharmacy (21.6%). Learning situation 1 has been held eight times since July 2021, situation 2 has been held three times since February 2023, and situation 3 has been held once since February 2024. Therefore, situation 1 has the highest number of participants. Respondents were recruited from 40 prefectures in Japan. The three most common prefectures are Tokyo, Osaka, and Kanagawa. Web-based participants came from 40 prefectures, whereas face-to-face participants came from 13 prefectures, including the two host cities of Tokyo and Osaka.

**Table 5:** Characteristics of respondents

		Total (*n* = 296)	Clinic (*n* = 5)	Hospital (*n* = 217)	Pharmacy (*n* = 74)	*p*-value
Sex	Female	167	4	127	36	0.214
	Male	129	1	90	38	
Age		42.1	51.2*	41.6*	42.9	0.033
Palliative drug therapy certified pharmacist	non-BCPPP	144	1	85	58	< 0.001
	BCPPP	152	4	132	16	
Training format	Web-based (10 times)	261	5	187	69	0.261
	Face-to-face (2 times)	35	0	30	5	
Learning situation	Scenario 1 (8 times)	226	3	171	52	0.0537
	Scenario 2 (3 times)	45	1	26	18	
	Scenario 3 (1 time)	25	1	20	4	

Sex, training format, case scenario, and certification status distributions were analyzed using Fisher’s exact test. Age was analyzed using one-way ANOVA. When significant differences were found, post hoc analyses were conducted using Tukey’s HSD test. Statistical significance was set at *p* < 0.05. *p < 0.05, based on Tukey’s HSD test

### Perspectives of respondents from the survey

Table 6 shows the results of the post-survey analyzed by facility affiliation (hospital, clinic or community pharmacy), training format (web-based or face-to-face), and certification (BCPPP and non-BCPPP). The overall usefulness of daily work was highly rated at 4.39 for Session I and 4.20 for Session II, suggesting that both sessions were useful in daily palliative care. No significant differences were noted in training format or palliative certification, but a significant difference was noted by facility affiliation in Session II, with community pharmacy pharmacists reporting slightly higher usefulness scores than hospital pharmacists. The overall difficulty level of the pSMILE workshop was rated 3.43 for Session I and 3.55 for Session II, suggesting that the participants found the sessions somewhat difficult. Session I was perceived as more difficult by community pharmacy pharmacists than by hospital pharmacists, with a significant difference (*p* < 0.001). By contrast, Session II was perceived as more difficult by hospital pharmacists than by community pharmacy pharmacists, with a significant difference (*p* < 0.001). In both Sessions I and II, certified-pharmacists (BCPPP) perceived significantly less difficulty than did non-certified pharmacists (non-BCPPP; *p* < 0.01). The overall length of the sessions was rated 2.66 for Session I and 2.42 for Session II, suggesting that the participants found the sessions to be somewhat short. In particular, Session II was perceived to be significantly shorter in web-based workshops than in face-to-face workshops; however, no differences were noted among facility affiliations. The average score for satisfaction with participation after the pre-assignment was 4.40, suggesting that participants were generally satisfied with completing the pre-assignment. No significant differences were found based on facility affiliation or training format. While both certified (BCPPP: 4.30) and non-certified (non-BCPPP: 4.50) participants reported high levels of satisfaction, non-BCPPP indicated significantly higher satisfaction than BCPPP (*p* = 0.033). The average score for overall satisfaction with the web-based training format was 4.26, with no differences in facility affiliation or certification. The average score for overall satisfaction with pSMILE was high (≥ 4.5), with no differences based on facility affiliation or certification. Regarding the training format, the average score for overall satisfaction with pSMILE was 4.47 for the web-based and 4.69 for the face-to-face workshop, suggesting that a comparable level of satisfaction was achieved even with the web-based workshop. Except for the length of Session II, no significant differences were noted in other items between the two formats, indicating that the web-based format provided a similar satisfactory experience as the face-to-face format.

**Table 6:** Assessment of pSMILE workshops based on post-participation questionnaires

	Total (*n* = 296)	Affiliation				Training Format			Palliative certification		
		Clinic (*n* = 5)	Hospital (*n* = 217)	Pharmacy (*n* = 74)	*p*-value	web-based (*n* = 261)	face-to-face (*n* = 35)	*p*-value	non-BCPPP (*n* = 144)	BCPPP (*n* = 152)	*p*-value
Session I: Usefulness in daily work	4.39 ± 0.59	4.20 ± 0.84	4.42 ± 0.58	4.30 ± 0.59	0.238	4.37 ± 0.60	4.49 ± 0.51	0.282	4.38 ± 0.61	4.39 ± 0.56	0.928
Session I: Difficulty level	3.43 ± 0.71	3.60 ± 0.55	3.28 ± 0.63*	3.86 ± 0.75*	< 0.001	3.43 ± 0.72	3.43 ± 0.56	0.973	3.65 ± 0.71	3.22 ± 0.63	< 0.001
Session I: Duration	2.66 ± 0.59	2.20 ± 0.45	2.65 ± 0.58	2.74 ± 0.62	0.101	2.65 ± 0.61	2.77 ± 0.43	0.247	2.72 ± 0.60	2.61 ± 0.59	0.091
Session II: Usefulness in daily work	4.20 ± 0.61	4.40 ± 0.55	4.14 ± 0.61*	4.35 ± 0.61*	0.026	4.20 ± 0.61	4.14 ± 0.65	0.586	4.14 ± 0.60	4.25 ± 0.62	0.119
Session II: Difficulty level	3.55 ± 0.81	3.00 ± 0.00	3.67 ± 0.71*	3.22 ± 1.00*	< 0.001	3.52 ± 0.82	3.77 ± 0.73	0.083	3.67 ± 0.94	3.43 ± 0.66	0.009
Session II: Duration	2.42 ± 0.70	2.20 ± 0.84	2.42 ± 0.70	2.43 ± 0.70	0.773	2.36 ± 0.70	2.86 ± 0.55	< 0.001	2.42 ± 0.72	2.41 ± 0.69	0.911
Satisfaction with the pre-assignment	4.40 ± 0.80	5.00 ± 0.00	4.38 ± 0.80	4.41 ± 0.81	0.23	4.39 ± 0.80	4.46 ± 0.74	0.644	4.50 ± 0.70	4.30 ± 0.87	0.033
Satisfaction with web-based Training format^a^	4.26 ± 0.93	3.60 ± 1.14	4.25 ± 0.95	4.33 ± 0.85	0.224	-	–	–	4.31 ± 0.88	4.21 ± 0.97	0.357
pSMILE overall satisfaction	4.50 ± 0.72	4.80 ± 0.45	4.46 ± 0.73	4.58 ± 0.70	0.298	4.47 ± 0.75	4.69 ± 0.47	0.099	4.57 ± 0.64	4.43 ± 0.79	0.091

One-way ANOVA was used to analyze the assessment of post-participation questionnaires by facility affiliation (clinic, hospital, and pharmacy). When significant differences were found, post hoc analyses were conducted using Tukey’s HSD test. Scores between training formats (web-based vs. face-to-face) and between certified (BCPPP) and non-certified (non-BCPPP) participants were analyzed using *t*-tests. Statistical significance was set at *p* < 0.05. ^a^Number of participants differed from that of other items (total = 261, clinic = 5, hospital = 187, pharmacy = 69, non-BCPPP = 125, and BCPPP = 136); *p < 0.05, based on Tukey’s HSD test

### Behavioral changes in daily palliative care following pSMILE workshop

**Fig. 1:**
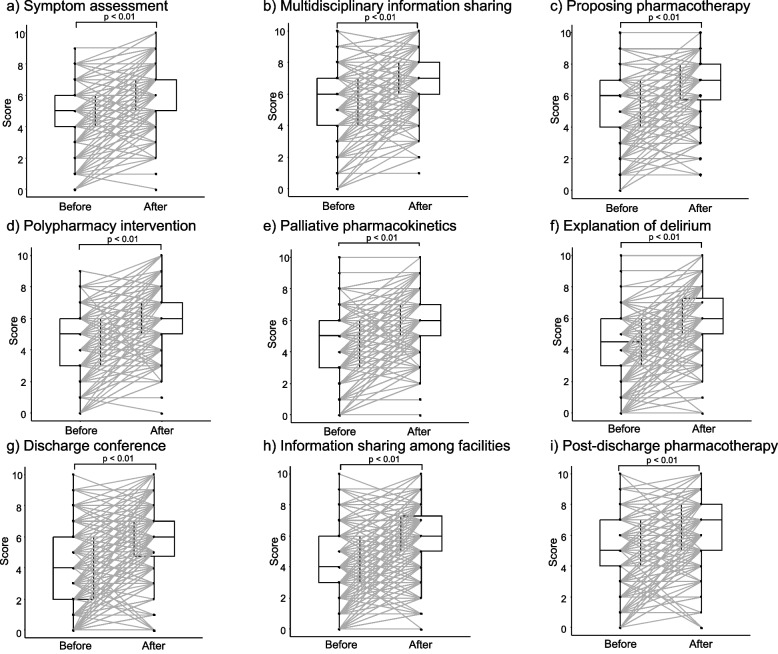
Behavioral changes before and 1 month after pSMILE workshop. **a** Symptom assessment; **b** multidisciplinary information sharing; **c** proposing pharmacotherapy to the attending physician; **d** polypharmacy intervention for patients undergoing palliative treatment; **e** palliative pharmacotherapy practice with awareness of pharmacokinetics; **f** explanation of delirium symptoms to patients and their families; **g** response at the time of participation in the discharge conference; **h** information sharing between hospitals, clinics, and community pharmacies; **i** suggestions for pharmacotherapy in view of the post-discharge period. Values are presented as median (interquartile range). Statistical significance was determined using the paired *t*-test

Figure 1 shows the changes in the confidence scores of behaviors related to daily palliative care before and 1 month after the workshop. All nine items (a: symptom assessment; b: multidisciplinary information sharing; c: proposing pharmacotherapy to the attending physician; d: polypharmacy intervention for patients undergoing palliative treatment; e: palliative pharmacotherapy practice with awareness of pharmacokinetics; f: explanation of delirium symptoms to patients and their families; g: response at the time of participation in the discharge conference; h: information sharing between hospitals, clinics, and community pharmacies; and i: suggestions for pharmacotherapy in view of the post-discharge period) increased significantly after the workshop (*p* < 0.001). Table 7 shows the results of the behavioral changes analyzed according to facility affiliation (hospital, clinic, or community pharmacy), training format (web-based or face-to-face), and palliative certification (BCPPP and non-BCPPP). Improvements in palliative care-related behaviors were observed regardless of facility affiliation, training format, or certification, indicating that behavioral changes were effectively achieved. By facility affiliation (clinic, hospital, or community pharmacy), all confidence scores (a–i) of daily palliative behaviors in hospital and community pharmacy pharmacists significantly improved following the workshop. However, no significant differences were noted between clinical pharmacists before and after the workshop, probably because of the small number of cases. Almost no differences were noted in scores among facility affiliations before or after the workshop. Confidence in proposing pharmacotherapy to the attending physician (c) was consistently higher in hospital pharmacists than in community pharmacy pharmacists both before and after the workshop. In contrast, before the workshop, confidence in information sharing between hospitals, clinics, and community pharmacies (h) was higher in clinic and community pharmacy pharmacists than in hospital pharmacists, but this difference was attenuated and no longer statistically significant after the workshop. Similarly, all confidence scores (a–i) for daily palliative behaviors in both the web-based and face-to-face workshops significantly improved after the workshop. Scores were nearly identical between the web-based and face-to-face workshops before the workshop, and no differences were observed afterward. Regarding certification, all confidence scores of both non-BCPPP and BCPPP significantly improved following the workshop. However, all scores of the BCPPP were significantly higher than those of the non-BCPPP before and after the workshop.

**Table 7:** Daily palliative behavior scores before and 1-month after workshop by affiliation, training format, and certification

	Facility affiliation									Training format						Palliative certification					
	Clinic (*n* = 5)			Hospital (*n* = 216)			Pharmacy (*n* = 71)			Web-based (*n* = 257)			Face-to-face (*n* = 35)			Non-BCPPP (*n* = 141)			BCPPP (*n* = 151)		
	Before	After	*p*	Before	After	*p*	Before	After	*p*	Before	After	*p*	Before	After	*p*	Before	After	*p*	Before	After	*p*
a	4.40 ± 1.95	5.40 ± 1.52	0.034	4.98 ± 1.92	6.44 ± 1.50^c^	< 0.01	4.35 ± 2.07	5.77 ± 2.05^c^	< 0.01	4.82 ± 2.00	6.25 ± 1.71	< 0.01	4.77 ± 1.80	6.31 ± 1.39	< 0.01	4.03 ± 1.96	5.70 ± 1.74	< 0.01	5.56 ± 1.67^e^	6.78 ± 1.42^f^	< 0.01
b	5.80 ± 1.92	6.60 ± 1.67	0.242	5.59 ± 1.97	6.70 ± 1.55	< 0.01	5.55 ± 2.41	7.06 ± 2.08	< 0.01	5.60 ± 2.08	6.79 ± 1.71	< 0.01	5.46 ± 2.08	6.74 ± 1.56	< 0.01	4.96 ± 2.20	6.42 ± 1.87	< 0.01	6.17 ± 1.78^e^	7.13 ± 1.43^f^	< 0.01
c	5.60 ± 1.67	6.00 ± 1.87	0.477	5.62 ± 1.97^a^	6.69 ± 1.64^c^	< 0.01	4.44 ± 2.03^a^	5.94 ± 1.96^c^	< 0.01	5.32 ± 2.06	6.47 ± 1.77	< 0.01	5.46 ± 1.95	6.71 ± 1.60	< 0.01	4.45 ± 2.08	5.84 ± 1.89	< 0.01	6.17 ± 1.61^e^	7.11 ± 1.36^f^	< 0.01
d	4.00 ± 2.24	5.40 ± 0.89	0.108	4.74 ± 1.96	6.19 ± 1.66	< 0.01	4.61 ± 2.07	6.08 ± 2.18	< 0.01	4.77 ± 1.99	6.14 ± 1.81	< 0.01	4.14 ± 1.93	6.29 ± 1.67	< 0.01	4.15 ± 2.03	5.72 ± 1.99	< 0.01	5.21 ± 1.81^e^	6.56 ± 1.48^f^	< 0.01
e	4.40 ± 1.52	5.40 ± 1.52	0.034	4.76 ± 1.94^a^	6.08 ± 1.67	< 0.01	3.69 ± 2.14^a^	5.58 ± 2.00	< 0.01	4.54 ± 2.03	5.97 ± 1.78	< 0.01	4.17 ± 2.01	5.80 ± 1.61	< 0.01	3.67 ± 2.05	5.45 ± 1.83	< 0.01	5.27 ± 1.68^e^	6.41 ± 1.56^f^	< 0.01
f	5.40 ± 2.30	6.40 ± 1.82	0.034	4.23 ± 2.13	5.80 ± 2.02	< 0.01	4.18 ± 2.17	6.28 ± 2.04	< 0.01	4.35 ± 2.15	5.97 ± 2.03	< 0.01	3.40 ± 1.90^d^	5.63 ± 2.00	< 0.01	3.45 ± 2.14	5.45 ± 2.22	< 0.01	4.98 ± 1.87^e^	6.37 ± 1.72^f^	< 0.01
g	5.80 ± 2.17	6.80 ± 1.92	0.189	3.92 ± 2.43	5.52 ± 2.19	< 0.01	4.63 ± 2.66	5.90 ± 2.43	< 0.01	4.14 ± 2.54	5.61 ± 2.30	< 0.01	4.00 ± 2.26	5.83 ± 1.87	< 0.01	3.55 ± 2.58	5.10 ± 2.30	< 0.01	4.66 ± 2.32^e^	6.14 ± 2.08^f^	< 0.01
h	6.40 ± 1.52^b^	6.80 ± 1.92	0.541	3.98 ± 2.27^ab^	5.73 ± 2.11	< 0.01	4.97 ± 2.17^a^	6.32 ± 2.12	< 0.01	4.32 ± 2.26	5.93 ± 2.12	< 0.01	3.89 ± 2.51	5.60 ± 2.19	< 0.01	3.82 ± 2.32	5.42 ± 2.21	< 0.01	4.68 ± 2.19^e^	6.34 ± 1.94^f^	< 0.01
i	5.40 ± 2.30	6.40 ± 2.41	0.142	4.98 ± 2.10	6.40 ± 1.74	< 0.01	5.07 ± 2.31	6.52 ± 2.33	< 0.01	5.06 ± 2.19	6.42 ± 1.95	< 0.01	4.63 ± 1.85	6.51 ± 1.58	< 0.01	4.30 ± 2.26	5.94 ± 2.13	< 0.01	5.67 ± 1.82^e^	6.88 ± 1.54^f^	< 0.01

The scores for daily palliative behaviors before and after the workshop were analyzed using paired* t*-tests. Scores for facility affiliation (clinic, hospital, and pharmacy) before and after the workshop were analyzed using one-way ANOVA, followed by the post hoc Tukey’s HSD test. Scores between training formats (web-based vs. face-to-face) and palliative certification (non-certified (non-BCPPP) vs. certified (BCPPP) participants) before and after the workshop were analyzed using *t*-tests. Statistical significance was set at p < 0.05. ^a,b^ *p* < 0.05 vs. facility affiliation before the workshop; ^c^ *p* < 0.05 vs. facility affiliation after the workshop; ^d^* p* < 0.05 vs. web-based before the workshop; ^e^ *p* < 0.01 vs. non-BCPPP before the workshop; ^f^ *p* < 0.01 vs. non-BCPPP after the workshop

## Discussion

The present data show that attending pSMILE training leads participants to change their behavior regardless of facility affiliation or certification, contributing to quality improvement in daily palliative care. Furthermore, web-based workshops achieved behavioral changes comparable to those of face-to-face workshops. The pSMILE training program, offered in both web-based and face-to-face, is effective for the sustainable education of palliative care pharmacists in hospitals and clinics/community pharmacies.

For over a year, we discussed the possibility of organizing a training course on communication even during the Covid-19 pandemic [14]. Several reports have demonstrated the usefulness of webinar educational programs provided by pharmacists [15, 16]. However, to the best of our knowledge, the pSMILE training program was the first attempt at organizing a web-based workshop with group discussions for pharmacists approved by an academic society. The usefulness of face-to-face workshops has already been proven in clinical research learning methods [17], while several issues have been identified in web-based group training. Unlike lecture-based listening, group discussions involve sensing other’s facial expressions and pauses [14]. In addition, limited familiarity with Zoom, challenges in managing group discussions, and technical issues require careful consideration. Committee members held numerous discussions and trial sessions to address these challenges. Over the 3 years since the first pSMILE workshop, we have continued to share and address these problems. Even after the Covid-19 pandemic was calming down and face-to-face events became possible, we continued to conduct web-based pSMILE workshops according to the requests of many participants. In the participant survey, the most common positive aspect of web-based workshops was the reduction in travel time and costs. Contrastingly, the best aspect of face-to-face workshops was the opportunity to chat and get to know all other participants, including the facilitators. Despite the advantages of each training format, the present results clearly show that web-based workshops could achieve behavioral changes with no difference from face-to-face workshops, indicating comparable effectiveness and usefulness, and both were highly satisfactory to the participants. Therefore, we believe that the most effective educational program would be to provide both web-based and face-to-face workshops in various situations to meet the need of each participant.

The pSMILE workshop consisted of two main sessions, the first half taking place in hospitals and the second half in community pharmacies or clinics. The present results revealed that the perception of difficulty differed between the first and second sessions depending on facility affiliation. In the pSMILE workshop, we grouped both pharmacists, which allowed the hospital pharmacists to lead the group discussion in the first session and the community pharmacy or clinic pharmacists to lead in the second session. As a result, participants gained insights that they could not gain on their own, and a learning cycle was created. Both pharmacists considered the experiences of teaching and being taught by each other highly satisfying. It has been reported that when community and hospital pharmacists are trained in the same hospital, both contribute to the patient care plan and their interventions often overlap. However, parenteral drug interventions and optimization routes of administration are mainly provided by hospital pharmacists, whereas community pharmacy pharmacists often intervene to improve adherence and drug discontinuation [18]. The pSMILE workshop deepened learning by bringing pharmacists from hospitals, community pharmacies, and clinics together for training. Bridging the gap between inpatient and outpatient settings should be considered to further optimize palliative care [18]. The pSMILE training program was considered consistent with this purpose.

Certified pharmacists (BCPPP) are more actively involved in palliative care than non-certified pharmacists (non-BCPPP) and can provide better care to improve patient quality of life [19] and higher medical economic benefits [20, 21]. Approximately half of the participants in the pSMILE workshop were certified pharmacists (BCPPP). Pharmacists with little experience in palliative care can learn to model daily clinical practice through discussions with pharmacists who hold certification (BCPPP). They often facilitated group discussions and provided support that enabled participants to express their opinions. Through such discussions, the training aims to foster mutual respect, professional drug knowledge and skills, as well as strong communication skills. In this study, non-BCPPP perceived pSMILE training to be more difficult than the BCPPP; however, their satisfaction levels were similarly high. Therefore, participation in the pSMILE program is a valuable learning opportunity for both BCPPP and non-BCPPP.

In this study, we evaluated the effectiveness of a training program by focusing on behavioral changes. The present results suggest that all confidence scores of daily palliative behaviors (symptom assessment; multidisciplinary information sharing; proposing pharmacotherapy to the attending physician; polypharmacy intervention for patients undergoing palliative treatment; palliative pharmacotherapy practice with awareness of pharmacokinetics; explanation of delirium symptoms to patients and their families; response at the time of participation in the discharge conference; information sharing between hospitals, clinics, and community pharmacies; and suggestions for pharmacotherapy in view of the post-discharge period) increased following the pSMILE workshop. All these items are often observed in daily clinical practice in the field of palliative care. Regarding clinic pharmacists, the smaller increase in motivation may reflect their intermediate role between hospitals and community pharmacies, as they are often already involved in post-discharge pharmacotherapy and care coordination. However, the sample size was small (*n* = 5), making it difficult to draw definitive conclusions. The improvement in behavioral changes can be attributed to the fact that participants can imagine their own behavior through discussions with others in the workshop, which may lead to increased confidence. According to Bandura’s theory of self-efficacy and behavior [22], increased self-efficacy contributes to actual behavior (performance achievement) when both knowledge and self-efficacy increase. In this study, only self-reported confidence and behavioral motivation were assessed, whereas knowledge acquisition was not directly evaluated. However, the observed increase in participants’ confidence and motivation suggests the possibility of behavioral changes according to the theory. The transtheoretical model can be used to classify and understand the stages of behavioral change [23–25]. This model describes five phases—pre-contemplation, contemplation, preparation, action, and maintenance—and allows us to evaluate participants’ progress from initial stages toward sustained behavioral change. Participants may have been in the pre-contemplation or contemplation phase prior to their participation, which could be evaluated as the action phase, as a clear change in behavior was observed after 1 month. We consider that a future issue is the evaluation of progress up to the maintenance phase after 6 months. The value of the training would be further enhanced if it were maintained on an ongoing basis and if specific details of behavioral changes were disclosed to demonstrate its usefulness.

Training for the facilitators of the pSMILE workshop was introduced in 2022. Currently, there are 16 facilitators, including 9 new ones. Facilitators were selected from among past pSMILE participants. In addition to their experiences in the palliative care field, their leadership during group discussions on the day of the workshop was also considered. The selection process was discussed and finalized among the current facilitators. Facilitator training is conducted once a year to educate facilitators so they can become independent within a few sessions. New facilitators are initially paired with experienced ones and received careful support. Through this approach, we aim to create a cycle of education.

Several improvements could be considered to further optimize the effectiveness of pSMILE program. First, Session II of the web-based workshop was perceived as relatively short. Offering an extended version for participants who wish more discussion time may be beneficial. Second, participation by clinic pharmacists was limited; adjusting the program schedule to accommodate their working conditions may encourage higher engagement. Finally, an advanced version of the program for certified pharmacists (BCPPP) could provide more specialized content. The implementation of these improvements can be explored in future revisions through feedback from participants.

This study has several limitations. First, it has only been 3 years since the introduction of pSMILE, with 296 total participants and 152 of whom held a BCPPP, despite there being 865 certified pharmacists as of 2025. As only approximately 20% of the certified pharmacists (BCPPP) participated, the survey results may not necessarily represent their views. Second, it cannot be ruled out that participation was voluntary and targeted pharmacists with high motivation. Therefore, participants tended to have higher initial awareness or interest in palliative care, which may have introduced selection bias and potentially led to an overestimation of behavioral changes. Third, even if qualitative research is conducted with caution, the possibility that the results may contain bias owing to the subjectivity and value of the researcher cannot be ruled out. Self-reported outcomes are subject to the Hawthorne effect [26], whereby participants may attempt to present themselves in a better light. Fourth, this study only considered the pharmacists’ perspective and not the perspectives of doctors, nurses, and medical social workers involved in the palliative care team. Fifth, long-term observations of behavioral changes were not available. Sixth, this study did not directly assess participants’ knowledge levels and objective outcomes such as the number of interventions or patient-related effects. Therefore, the findings should be interpreted as preliminary evidence of self-reported improvements in motivation and confidence. In the future, we believe these limitations can be addresses through further research, such as developing questionnaires based on the categories and concepts identified in this study, and conducting a series of quantitative surveys.

## Conclusions

This is the first study to demonstrate the effectiveness and usefulness of an academically approved pSMILE educational program for palliative care pharmacists in both web-based and face-to-face workshops. The participants’ behavioral changes regarding palliative care improved after attending training, contributing to quality improvement in daily palliative care.

## Supplementary Information

Supplementary Material 1.

## Data Availability

All study data that support the findings of this study are available from the corresponding author, M.K, upon reasonable request.

## Abbreviations

pSMILE: Palliative care-Situational Motivating Interactive Learning and Education
BCPPP: Board Certified Pharmacist in Palliative Pharmacy
JPPS: Japanese Society for Pharmaceutical Palliative Care and Sciences
PEOPLE: Pharmacy Education for Oncology and Palliative care Leading to happy End-of-life
ASHP: American Society of Health-System Pharmacists
ANOVA: Analysis of variance
Tukey’s HSD: Tukey’s honest significant difference
