# Supplementary material for: Evaluation of the pSMILE (Palliative care-Situational Motivating Interactive Learning and Education) educational program based on web-based and face-to-face workshops for palliative care pharmacists
Source: J Pharm Health Care Sci. 2025 Dec 20;11:109. doi: 10.1186/s40780-025-00491-w (PMC12720441; doi:10.1186/s40780-025-00491-w)
Supplement: Supplementary file 1 — Supplementary Material 1. [file 40780_2025_491_MOESM1_ESM.docx]

Supplementary Table 1. Roles of Pharmacists in Palliative and Hospice Care (adapted from ASHP Guidelines)

| Roles | Role description | Examples of activities |
| --- | --- | --- |
| Essential | Provide patient-centered pharmacotherapy to optimize symptom management and palliative care outcomes | Review and adjust medications for pain, dyspnea, nausea; discontinue non-beneficial drugs |
|  | Serve as a resource for evidence-based medication use | Provide drug information to clinicians, recommend safe opioid rotation |
|  | Recommend, modify, or discontinue pharmacotherapy in preparation for transitions of care | Medication reconciliation at discharge, deprescribing unnecessary medications |
|  | Collaborate with patients, families, and the care team to reassess goals of therapy | Participate in family conferences, align pharmacotherapy with patient’s care goals |
|  | Ensure compliance with regulations and institutional standards | Monitor controlled substance use, ensure formulary adherence |
| Desirable | Provide leadership within the interdisciplinary palliative care team | Lead team discussions, contribute to care pathway design |
|  | Perform medication therapy management (MTM) and collaborative practice under agreements (CPA) | Adjust doses under collaborative prescribing protocols |
|  | Educate patients, families, and healthcare professionals | Conduct educational sessions on pain management and end-of-life pharmacotherapy |
|  | Contribute to institutional policy, quality improvement, and research | Develop clinical guidelines, engage in palliative care research |
